# Supplementary material for: COZOID: contact zone identifier for visual analysis of protein-protein interactions
Source: BMC Bioinformatics. 2018 Apr 6;19:125. doi: 10.1186/s12859-018-2113-6 (PMC5889581; doi:10.1186/s12859-018-2113-6)
Supplement: Supplementary file 2 — Software build. Executable binary file of the software tool. (ZIP 118,784 kb) [file 12859_2018_2113_MOESM2_ESM.zip › cozoid/platform/docs/testng-6.5.1-javadoc.zip!/org/testng/Assert.html]

Assert (TestNG)


---


|  |  |  |
| --- | --- | --- |
| **Overview** \| **Package** \| **Class** \| **Tree** \| **Deprecated** \| **Index** \| **Help** | |  |
| PREV CLASS   **NEXT CLASS** | **FRAMES**    **NO FRAMES**     **All Classes** |  |
| SUMMARY: NESTED \| FIELD \| CONSTR \| METHOD | DETAIL: FIELD \| CONSTR \| METHOD |  |


---


## org.testng Class Assert

```
java.lang.Object
  org.testng.Assert
```

---

``` public class Assert extends java.lang.Object ```

Assertion tool class. Presents assertion methods with a more natural parameter order.
The order is always **actualValue**, **expectedValue** [, message].

**Author:**
:   Alexandru Popescu

---

| **Constructor Summary** | |
| --- | --- |
| `protected` | `Assert()`             Protect constructor since it is a static only class |


| **Method Summary** | |
| --- | --- |
| `static void` | `assertEquals(boolean actual, boolean expected)`             Asserts that two booleans are equal. |
| `static void` | `assertEquals(boolean actual, boolean expected, java.lang.String message)`             Asserts that two booleans are equal. |
| `static void` | `assertEquals(byte[] actual, byte[] expected)`             Asserts that two arrays contain the same elements in the same order. |
| `static void` | `assertEquals(byte[] actual, byte[] expected, java.lang.String message)`             Asserts that two arrays contain the same elements in the same order. |
| `static void` | `assertEquals(byte actual, byte expected)`             Asserts that two bytes are equal. |
| `static void` | `assertEquals(byte actual, byte expected, java.lang.String message)`             Asserts that two bytes are equal. |
| `static void` | `assertEquals(char actual, char expected)`             Asserts that two chars are equal. |
| `static void` | `assertEquals(char actual, char expected, java.lang.String message)`             Asserts that two chars are equal. |
| `static void` | `assertEquals(java.util.Collection actual, java.util.Collection expected)`             Asserts that two collections contain the same elements in the same order. |
| `static void` | `assertEquals(java.util.Collection actual, java.util.Collection expected, java.lang.String message)`             Asserts that two collections contain the same elements in the same order. |
| `static void` | `assertEquals(double actual, double expected, double delta)`             Asserts that two doubles are equal concerning a delta. |
| `static void` | `assertEquals(double actual, double expected, double delta, java.lang.String message)`             Asserts that two doubles are equal concerning a delta. |
| `static void` | `assertEquals(float actual, float expected, float delta)`             Asserts that two floats are equal concerning a delta. |
| `static void` | `assertEquals(float actual, float expected, float delta, java.lang.String message)`             Asserts that two floats are equal concerning a delta. |
| `static void` | `assertEquals(int actual, int expected)`             Asserts that two ints are equal. |
| `static void` | `assertEquals(int actual, int expected, java.lang.String message)`             Asserts that two ints are equal. |
| `static void` | `assertEquals(long actual, long expected)`             Asserts that two longs are equal. |
| `static void` | `assertEquals(long actual, long expected, java.lang.String message)`             Asserts that two longs are equal. |
| `static void` | `assertEquals(java.util.Map<?,?> actual, java.util.Map<?,?> expected)`             Asserts that two maps are equal. |
| `static void` | `assertEquals(java.lang.Object[] actual, java.lang.Object[] expected)`             Asserts that two arrays contain the same elements in the same order. |
| `static void` | `assertEquals(java.lang.Object[] actual, java.lang.Object[] expected, java.lang.String message)`             Asserts that two arrays contain the same elements in the same order. |
| `static void` | `assertEquals(java.lang.Object actual, java.lang.Object expected)`             Asserts that two objects are equal. |
| `static void` | `assertEquals(java.lang.Object actual, java.lang.Object expected, java.lang.String message)`             Asserts that two objects are equal. |
| `static void` | `assertEquals(java.util.Set<?> actual, java.util.Set<?> expected, java.lang.String message)`             Assert set equals |
| `static void` | `assertEquals(java.util.Set actual, java.util.Set expected)`             Asserts that two sets are equal. |
| `static void` | `assertEquals(short actual, short expected)`             Asserts that two shorts are equal. |
| `static void` | `assertEquals(short actual, short expected, java.lang.String message)`             Asserts that two shorts are equal. |
| `static void` | `assertEquals(java.lang.String actual, java.lang.String expected)`             Asserts that two Strings are equal. |
| `static void` | `assertEquals(java.lang.String actual, java.lang.String expected, java.lang.String message)`             Asserts that two Strings are equal. |
| `static void` | `assertEqualsNoOrder(java.lang.Object[] actual, java.lang.Object[] expected)`             Asserts that two arrays contain the same elements in no particular order. |
| `static void` | `assertEqualsNoOrder(java.lang.Object[] actual, java.lang.Object[] expected, java.lang.String message)`             Asserts that two arrays contain the same elements in no particular order. |
| `static void` | `assertFalse(boolean condition)`             Asserts that a condition is false. |
| `static void` | `assertFalse(boolean condition, java.lang.String message)`             Asserts that a condition is false. |
| `static void` | `assertNotEquals(double actual1, double actual2, double delta)` |
| `static void` | `assertNotEquals(double actual1, double actual2, double delta, java.lang.String message)` |
| `static void` | `assertNotEquals(float actual1, float actual2, float delta)` |
| `static void` | `assertNotEquals(float actual1, float actual2, float delta, java.lang.String message)` |
| `static void` | `assertNotEquals(java.lang.Object actual1, java.lang.Object actual2)` |
| `static void` | `assertNotEquals(java.lang.Object actual1, java.lang.Object actual2, java.lang.String message)` |
| `static void` | `assertNotNull(java.lang.Object object)`             Asserts that an object isn't null. |
| `static void` | `assertNotNull(java.lang.Object object, java.lang.String message)`             Asserts that an object isn't null. |
| `static void` | `assertNotSame(java.lang.Object actual, java.lang.Object expected)`             Asserts that two objects do not refer to the same object. |
| `static void` | `assertNotSame(java.lang.Object actual, java.lang.Object expected, java.lang.String message)`             Asserts that two objects do not refer to the same objects. |
| `static void` | `assertNull(java.lang.Object object)`             Asserts that an object is null. |
| `static void` | `assertNull(java.lang.Object object, java.lang.String message)`             Asserts that an object is null. |
| `static void` | `assertSame(java.lang.Object actual, java.lang.Object expected)`             Asserts that two objects refer to the same object. |
| `static void` | `assertSame(java.lang.Object actual, java.lang.Object expected, java.lang.String message)`             Asserts that two objects refer to the same object. |
| `static void` | `assertTrue(boolean condition)`             Asserts that a condition is true. |
| `static void` | `assertTrue(boolean condition, java.lang.String message)`             Asserts that a condition is true. |
| `static void` | `fail()`             Fails a test with no message. |
| `static void` | `fail(java.lang.String message)`             Fails a test with the given message. |
| `static void` | `fail(java.lang.String message, java.lang.Throwable realCause)`             Fails a test with the given message and wrapping the original exception. |

**Methods inherited from class java.lang.Object**

`clone, equals, finalize, getClass, hashCode, notify, notifyAll, toString, wait, wait, wait`

**Constructor Detail**

### Assert

```
protected Assert()
```

:   Protect constructor since it is a static only class


**Method Detail**

### assertTrue

```
public static void assertTrue(boolean condition,
                              java.lang.String message)
```

:   Asserts that a condition is true. If it isn't,
    an AssertionError, with the given message, is thrown.

    :   **Parameters:**: `condition` - the condition to evaluate: `message` - the assertion error message

---


### assertTrue

```
public static void assertTrue(boolean condition)
```

:   Asserts that a condition is true. If it isn't,
    an AssertionError is thrown.

    :   **Parameters:**: `condition` - the condition to evaluate

---


### assertFalse

```
public static void assertFalse(boolean condition,
                               java.lang.String message)
```

:   Asserts that a condition is false. If it isn't,
    an AssertionError, with the given message, is thrown.

    :   **Parameters:**: `condition` - the condition to evaluate: `message` - the assertion error message

---


### assertFalse

```
public static void assertFalse(boolean condition)
```

:   Asserts that a condition is false. If it isn't,
    an AssertionError is thrown.

    :   **Parameters:**: `condition` - the condition to evaluate

---


### fail

```
public static void fail(java.lang.String message,
                        java.lang.Throwable realCause)
```

:   Fails a test with the given message and wrapping the original exception.

    :   **Parameters:**: `message` - the assertion error message: `realCause` - the original exception

---


### fail

```
public static void fail(java.lang.String message)
```

:   Fails a test with the given message.

    :   **Parameters:**: `message` - the assertion error message

---


### fail

```
public static void fail()
```

:   Fails a test with no message.

---


### assertEquals

```
public static void assertEquals(java.lang.Object actual,
                                java.lang.Object expected,
                                java.lang.String message)
```

:   Asserts that two objects are equal. If they are not,
    an AssertionError, with the given message, is thrown.

    :   **Parameters:**: `actual` - the actual value: `expected` - the expected value: `message` - the assertion error message

---


### assertEquals

```
public static void assertEquals(java.lang.Object actual,
                                java.lang.Object expected)
```

:   Asserts that two objects are equal. If they are not,
    an AssertionError is thrown.

    :   **Parameters:**: `actual` - the actual value: `expected` - the expected value

---


### assertEquals

```
public static void assertEquals(java.lang.String actual,
                                java.lang.String expected,
                                java.lang.String message)
```

:   Asserts that two Strings are equal. If they are not,
    an AssertionError, with the given message, is thrown.

    :   **Parameters:**: `actual` - the actual value: `expected` - the expected value: `message` - the assertion error message

---


### assertEquals

```
public static void assertEquals(java.lang.String actual,
                                java.lang.String expected)
```

:   Asserts that two Strings are equal. If they are not,
    an AssertionError is thrown.

    :   **Parameters:**: `actual` - the actual value: `expected` - the expected value

---


### assertEquals

```
public static void assertEquals(double actual,
                                double expected,
                                double delta,
                                java.lang.String message)
```

:   Asserts that two doubles are equal concerning a delta. If they are not,
    an AssertionError, with the given message, is thrown. If the expected
    value is infinity then the delta value is ignored.

    :   **Parameters:**: `actual` - the actual value: `expected` - the expected value: `delta` - the absolute tolerate value value between the actual and expected value: `message` - the assertion error message

---


### assertEquals

```
public static void assertEquals(double actual,
                                double expected,
                                double delta)
```

:   Asserts that two doubles are equal concerning a delta. If they are not,
    an AssertionError is thrown. If the expected value is infinity then the
    delta value is ignored.

    :   **Parameters:**: `actual` - the actual value: `expected` - the expected value: `delta` - the absolute tolerate value value between the actual and expected value

---


### assertEquals

```
public static void assertEquals(float actual,
                                float expected,
                                float delta,
                                java.lang.String message)
```

:   Asserts that two floats are equal concerning a delta. If they are not,
    an AssertionError, with the given message, is thrown. If the expected
    value is infinity then the delta value is ignored.

    :   **Parameters:**: `actual` - the actual value: `expected` - the expected value: `delta` - the absolute tolerate value value between the actual and expected value: `message` - the assertion error message

---


### assertEquals

```
public static void assertEquals(float actual,
                                float expected,
                                float delta)
```

:   Asserts that two floats are equal concerning a delta. If they are not,
    an AssertionError is thrown. If the expected
    value is infinity then the delta value is ignored.

    :   **Parameters:**: `actual` - the actual value: `expected` - the expected value: `delta` - the absolute tolerate value value between the actual and expected value

---


### assertEquals

```
public static void assertEquals(long actual,
                                long expected,
                                java.lang.String message)
```

:   Asserts that two longs are equal. If they are not,
    an AssertionError, with the given message, is thrown.

    :   **Parameters:**: `actual` - the actual value: `expected` - the expected value: `message` - the assertion error message

---


### assertEquals

```
public static void assertEquals(long actual,
                                long expected)
```

:   Asserts that two longs are equal. If they are not,
    an AssertionError is thrown.

    :   **Parameters:**: `actual` - the actual value: `expected` - the expected value

---


### assertEquals

```
public static void assertEquals(boolean actual,
                                boolean expected,
                                java.lang.String message)
```

:   Asserts that two booleans are equal. If they are not,
    an AssertionError, with the given message, is thrown.

    :   **Parameters:**: `actual` - the actual value: `expected` - the expected value: `message` - the assertion error message

---


### assertEquals

```
public static void assertEquals(boolean actual,
                                boolean expected)
```

:   Asserts that two booleans are equal. If they are not,
    an AssertionError is thrown.

    :   **Parameters:**: `actual` - the actual value: `expected` - the expected value

---


### assertEquals

```
public static void assertEquals(byte actual,
                                byte expected,
                                java.lang.String message)
```

:   Asserts that two bytes are equal. If they are not,
    an AssertionError, with the given message, is thrown.

    :   **Parameters:**: `actual` - the actual value: `expected` - the expected value: `message` - the assertion error message

---


### assertEquals

```
public static void assertEquals(byte actual,
                                byte expected)
```

:   Asserts that two bytes are equal. If they are not,
    an AssertionError is thrown.

    :   **Parameters:**: `actual` - the actual value: `expected` - the expected value

---


### assertEquals

```
public static void assertEquals(char actual,
                                char expected,
                                java.lang.String message)
```

:   Asserts that two chars are equal. If they are not,
    an AssertionFailedError, with the given message, is thrown.

    :   **Parameters:**: `actual` - the actual value: `expected` - the expected value: `message` - the assertion error message

---


### assertEquals

```
public static void assertEquals(char actual,
                                char expected)
```

:   Asserts that two chars are equal. If they are not,
    an AssertionError is thrown.

    :   **Parameters:**: `actual` - the actual value: `expected` - the expected value

---


### assertEquals

```
public static void assertEquals(short actual,
                                short expected,
                                java.lang.String message)
```

:   Asserts that two shorts are equal. If they are not,
    an AssertionFailedError, with the given message, is thrown.

    :   **Parameters:**: `actual` - the actual value: `expected` - the expected value: `message` - the assertion error message

---


### assertEquals

```
public static void assertEquals(short actual,
                                short expected)
```

:   Asserts that two shorts are equal. If they are not,
    an AssertionError is thrown.

    :   **Parameters:**: `actual` - the actual value: `expected` - the expected value

---


### assertEquals

```
public static void assertEquals(int actual,
                                int expected,
                                java.lang.String message)
```

:   Asserts that two ints are equal. If they are not,
    an AssertionFailedError, with the given message, is thrown.

    :   **Parameters:**: `actual` - the actual value: `expected` - the expected value: `message` - the assertion error message

---


### assertEquals

```
public static void assertEquals(int actual,
                                int expected)
```

:   Asserts that two ints are equal. If they are not,
    an AssertionError is thrown.

    :   **Parameters:**: `actual` - the actual value: `expected` - the expected value

---


### assertNotNull

```
public static void assertNotNull(java.lang.Object object)
```

:   Asserts that an object isn't null. If it is,
    an AssertionError is thrown.

    :   **Parameters:**: `object` - the assertion object

---


### assertNotNull

```
public static void assertNotNull(java.lang.Object object,
                                 java.lang.String message)
```

:   Asserts that an object isn't null. If it is,
    an AssertionFailedError, with the given message, is thrown.

    :   **Parameters:**: `object` - the assertion object: `message` - the assertion error message

---


### assertNull

```
public static void assertNull(java.lang.Object object)
```

:   Asserts that an object is null. If it is not,
    an AssertionError, with the given message, is thrown.

    :   **Parameters:**: `object` - the assertion object

---


### assertNull

```
public static void assertNull(java.lang.Object object,
                              java.lang.String message)
```

:   Asserts that an object is null. If it is not,
    an AssertionFailedError, with the given message, is thrown.

    :   **Parameters:**: `object` - the assertion object: `message` - the assertion error message

---


### assertSame

```
public static void assertSame(java.lang.Object actual,
                              java.lang.Object expected,
                              java.lang.String message)
```

:   Asserts that two objects refer to the same object. If they do not,
    an AssertionFailedError, with the given message, is thrown.

    :   **Parameters:**: `actual` - the actual value: `expected` - the expected value: `message` - the assertion error message

---


### assertSame

```
public static void assertSame(java.lang.Object actual,
                              java.lang.Object expected)
```

:   Asserts that two objects refer to the same object. If they do not,
    an AssertionError is thrown.

    :   **Parameters:**: `actual` - the actual value: `expected` - the expected value

---


### assertNotSame

```
public static void assertNotSame(java.lang.Object actual,
                                 java.lang.Object expected,
                                 java.lang.String message)
```

:   Asserts that two objects do not refer to the same objects. If they do,
    an AssertionError, with the given message, is thrown.

    :   **Parameters:**: `actual` - the actual value: `expected` - the expected value: `message` - the assertion error message

---


### assertNotSame

```
public static void assertNotSame(java.lang.Object actual,
                                 java.lang.Object expected)
```

:   Asserts that two objects do not refer to the same object. If they do,
    an AssertionError is thrown.

    :   **Parameters:**: `actual` - the actual value: `expected` - the expected value

---


### assertEquals

```
public static void assertEquals(java.util.Collection actual,
                                java.util.Collection expected)
```

:   Asserts that two collections contain the same elements in the same order. If they do not,
    an AssertionError is thrown.

    :   **Parameters:**: `actual` - the actual value: `expected` - the expected value

---


### assertEquals

```
public static void assertEquals(java.util.Collection actual,
                                java.util.Collection expected,
                                java.lang.String message)
```

:   Asserts that two collections contain the same elements in the same order. If they do not,
    an AssertionError, with the given message, is thrown.

    :   **Parameters:**: `actual` - the actual value: `expected` - the expected value: `message` - the assertion error message

---


### assertEquals

```
public static void assertEquals(java.lang.Object[] actual,
                                java.lang.Object[] expected,
                                java.lang.String message)
```

:   Asserts that two arrays contain the same elements in the same order. If they do not,
    an AssertionError, with the given message, is thrown.

    :   **Parameters:**: `actual` - the actual value: `expected` - the expected value: `message` - the assertion error message

---


### assertEqualsNoOrder

```
public static void assertEqualsNoOrder(java.lang.Object[] actual,
                                       java.lang.Object[] expected,
                                       java.lang.String message)
```

:   Asserts that two arrays contain the same elements in no particular order. If they do not,
    an AssertionError, with the given message, is thrown.

    :   **Parameters:**: `actual` - the actual value: `expected` - the expected value: `message` - the assertion error message

---


### assertEquals

```
public static void assertEquals(java.lang.Object[] actual,
                                java.lang.Object[] expected)
```

:   Asserts that two arrays contain the same elements in the same order. If they do not,
    an AssertionError is thrown.

    :   **Parameters:**: `actual` - the actual value: `expected` - the expected value

---


### assertEqualsNoOrder

```
public static void assertEqualsNoOrder(java.lang.Object[] actual,
                                       java.lang.Object[] expected)
```

:   Asserts that two arrays contain the same elements in no particular order. If they do not,
    an AssertionError is thrown.

    :   **Parameters:**: `actual` - the actual value: `expected` - the expected value

---


### assertEquals

```
public static void assertEquals(byte[] actual,
                                byte[] expected)
```

:   Asserts that two arrays contain the same elements in the same order. If they do not,
    an AssertionError is thrown.

    :   **Parameters:**: `actual` - the actual value: `expected` - the expected value

---


### assertEquals

```
public static void assertEquals(byte[] actual,
                                byte[] expected,
                                java.lang.String message)
```

:   Asserts that two arrays contain the same elements in the same order. If they do not,
    an AssertionError, with the given message, is thrown.

    :   **Parameters:**: `actual` - the actual value: `expected` - the expected value: `message` - the assertion error message

---


### assertEquals

```
public static void assertEquals(java.util.Set actual,
                                java.util.Set expected)
```

:   Asserts that two sets are equal.

---


### assertEquals

```
public static void assertEquals(java.util.Set<?> actual,
                                java.util.Set<?> expected,
                                java.lang.String message)
```

:   Assert set equals

---


### assertEquals

```
public static void assertEquals(java.util.Map<?,?> actual,
                                java.util.Map<?,?> expected)
```

:   Asserts that two maps are equal.

---


### assertNotEquals

```
public static void assertNotEquals(java.lang.Object actual1,
                                   java.lang.Object actual2,
                                   java.lang.String message)
```

---


### assertNotEquals

```
public static void assertNotEquals(java.lang.Object actual1,
                                   java.lang.Object actual2)
```

---


### assertNotEquals

```
public static void assertNotEquals(float actual1,
                                   float actual2,
                                   float delta,
                                   java.lang.String message)
```

---


### assertNotEquals

```
public static void assertNotEquals(float actual1,
                                   float actual2,
                                   float delta)
```

---


### assertNotEquals

```
public static void assertNotEquals(double actual1,
                                   double actual2,
                                   double delta,
                                   java.lang.String message)
```

---


### assertNotEquals

```
public static void assertNotEquals(double actual1,
                                   double actual2,
                                   double delta)
```


---


|  |  |  |
| --- | --- | --- |
| **Overview** \| **Package** \| **Class** \| **Tree** \| **Deprecated** \| **Index** \| **Help** | |  |
| PREV CLASS   **NEXT CLASS** | **FRAMES**    **NO FRAMES**     **All Classes** |  |
| SUMMARY: NESTED \| FIELD \| CONSTR \| METHOD | DETAIL: FIELD \| CONSTR \| METHOD |  |


---
